# How the interrelated physical, social and organizational environment impacts daily life of residents with dementia on a Green Care Farm

**DOI:** 10.3389/fpubh.2022.946962

**Published:** 2022-08-29

**Authors:** Katharina Rosteius, Bram de Boer, Sandra Staudacher, Jos Schols, Hilde Verbeek

**Affiliations:** ^1^Department of Health Services Research, Care and Public Health Research Institute, Faculty of Health, Medicine and Life Sciences, Maastricht University, Maastricht, Netherlands; ^2^Living Lab in Ageing and Long Term Care, Maastricht, Netherlands; ^3^Department of Public Health, Faculty of Medicine, Institute of Nursing Science, University of Basel, Basel, Switzerland

**Keywords:** dementia, innovative nursing home, long-term care, Green Care Farm, leadership, residents, meaningful activities

## Abstract

Green Care Farms (GCF) are innovative long-term care environments and an alternative to regular nursing homes in the Netherlands. Following a culture change movement, GCFs have radically altered the care environment. Research suggests positive effects on residents. However, knowledge is limited regarding their physical, social and organizational environment. This article explores the care environment of 24-h GCFs for people with dementia and its impact on residents and their daily life. An ethnographic study using mixed methods was carried out at a GCF in the Netherlands between June and October 2021. Researchers lived on the GCF and completed 28 days of participatory observations in three groups. During the day, informal conversations were held with residents (*n* = 48), staff and family members. Twenty four semi-structured interviews were conducted with residents, their family members, staff and the managers, complemented by a focus group with staff. The physical environment was additionally assessed with the OAZIS-dementia tool. Data collection methods informed each other. Qualitative data was thematically analyzed, quantitative data descriptively. Four themes were identified as crucial during daily life on the GCF: stimulating the senses, engaging in purposeful activities, sharing responsibilities and creating a community in a new home. Realizing these topics in practice, physical, social and organizational environmental components were highly interrelated. The physical environment encouraged and facilitated meaningful in-/outdoor activities and social encounters. The organizational environment supported the use of the physical environment by aligning processes and transporting the vision. The social environment focused on collaboration and creating a home-like atmosphere by including residents in household- and farm chores. This community-building led to more meaningful activities and social interaction. In conclusion, this study revealed the central influence of the management in paving the way for a new form of care delivery. As leaders shape the three environments, the organization influences the design of the physical environment and the actions taking place within it. By creating a community, the care home benefits residents, their families and staff equally. The conscious interrelation and harmonization of the physical, social and organizational components of a long-term care environment has the potential to improve the daily life of residents.

## Introduction

Due to the continuous aging of the Western societies, age-related diseases are on the rise, especially neurodegenerative conditions like dementia (1, 2). The simultaneous increase in care demands and decrease in human and financial resources calls for a different approach of organizing care and support for those in need of long-term care (3, 4). Traditional long-term care facilities are often based on a medical understanding of long-term care (5). Evidence suggests high levels of inactivity (6) and neuropsychiatric symptoms (7), as well as a high use of psychotropic drugs (8) in people living in traditional long-term care facilities. Following a culture change in long-term care, innovative concepts have been introduced, delivering care to vulnerable older people in smaller, more home-like environments than traditional larger long-term care facilities. Based on a more psychosocial understanding of long-term care, care is evolving around autonomy, maintaining daily functioning and sustainably engaging in meaningful activities with a focus on well-being (9).

One of these innovative initiatives are Green Care Farms (GCF), which are among the fastest growing forms of multifunctional agriculture (10). GCFs not only employ a different care vision, they also actively incorporate natural activities into the daily life. Examples include caring for animals, working in the garden, or cooking with homegrown vegetables (11, 12). The care focuses on stimulating self-reliance and offering a meaningful daytime activity, which might help people with dementia to stay active for a longer time (13). Research also indicates that residents at GCFs are more active than residents in traditional settings and are more physically and socially engaged during activities carried out (14). Furthermore, studies have found positive effects of day care at GCFs on dietary intake of people living with dementia (15). These positive effects can be linked to the radically different care environment of GCFs.

The care environment plays a crucial role in the progress of people with dementia and can both hinder or support their physical, mental and social functioning (16). Each care environment has physical, social and organizational features, each influencing the way, care is delivered (13). The physical environment is the tangible environment with natural and human-made objects. It can be a barrier or an enabler for people (17). The built environment can support purposeful activity and quality of life, especially for people with dementia (18, 19). Examples include the design of the indoor and outdoor environment, the privacy of rooms or the furnishing of communal areas. The social environment describes the social setting in which people live or act (20). It is comprised of human contacts, stimulation, activities (21), but also the larger cultural values (22). An example is relationship-centered care, which aims to involve the social network of a person into care (23). Lastly, the organizational environment describes not only the structure of an organization, but also the processes (24). A structural element could be the division of tasks, while rules or routines that guide staff actions are company-specific processes. Shared values and a supportive leadership, for example, have been found to improve the delivery of care (25).

Alternative care concepts like CGFs have radically changed the physical, social and organizational environment to better meet the needs of residents, their family members and staff (13). They are part of a culture change movement toward more suitable living environments for people with care needs and a more age-friendly society. By providing care focusing on the person and their relational context, not the disability, such concepts can provide other, more traditional care facilities with valuable insights on how to rethink dementia care. Traditional care organizations aiming to redesign their care delivery often face difficulties in implementing change [e.g., (26)]. Bound to existing buildings, but also routines and regulations, the implementation of a new vision on care often proves to be challenging (27). Therefore, practical knowledge is needed on innovative care environments such as GCFs, providing other care organizations with examples on how to sustainably and successfully implement changes that benefit all stakeholders involved. Although GCFs are becoming a more prominent alternative to regular care, there is little knowledge on the underlying components and working mechanisms of this innovative care environment. Therefore, the aim of this study is to analyze the care environment of GCFs based on their physical, social and organizational context.

## Methods

### Design

An explorative, mixed-methods ethnographic case study was conducted between May 2021 and October 2021. Aiming to understand the way in which care is delivered at GCFs, as well as opinions and experiences of involved stakeholders, this study took a constructivist perspective (28).

### Setting

**Figure 1:**
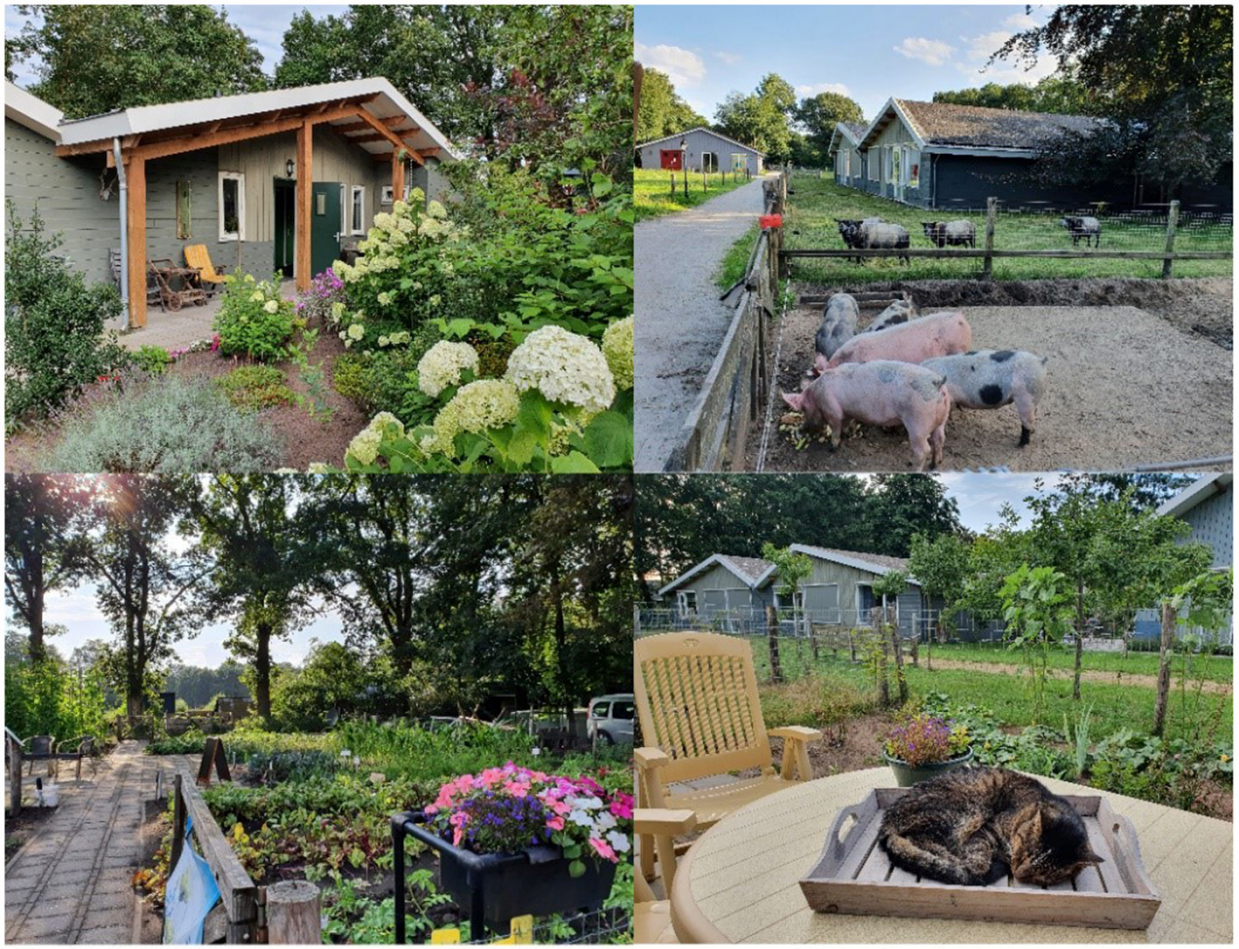
Illustrative images of the Dutch Green Care Farm “ZorgErf buiten-verbijf”.

The study took place at the privately owned GCF “ZorgErf buiten-verblijf” in the Netherlands, newly built in 2014 (see Figure 1 for illustrative images). ZorgErf is officially registered as care home, focusing on people with dementia only. Admission is based on official Dutch regulations considering the care dependency level. The care, to which a person is entitled to, is determined by a standardized procedure, carried out by a government agency (29).

The GCF is located in the countryside, not far from a small city. It has 48 rooms available for people living with dementia, which are organized in three groups. In each group, 16 residents live in small houses accessible through a garden surrounding a large common house. Each common house has two living rooms and a kitchen on the ground floor, and an office, as well as a small meeting room on the first floor. The entire common house is furnished in a homelike manner, often with furniture and art from residents themselves. The groups are mostly self-organized regarding daily life. This includes for example the planning, ordering and preparing of all meals or the determination of the daily activities and the time-schedule. The GCF has an open-door policy, allowing residents to freely access the entire 3-acre location. Here, they can visit vegetable gardens and several animals such as chicken, horses, pigs or sheep. The facilities include a country house, where various events take place and a café with a large terrace is included. Furthermore, a day-care for around 30 guests per day is part of the location; however, it was not focus of this study.

During the time of the study, the staff of each group consisted of registered nurses, certified nurses, nursing assistants and hostesses. During daytime, two care staff members and one hostess were permanently present in each group, supported by two shorter stays of hostesses during midday and the evening. At night, two care staff members were present for the entire location. Often, interns or volunteers supplemented staffing levels, and during times of more complex care situations, more staff hours were possible.

### Data collection

All data was collected from June 2021–October 2021. Four types of data collection methods were used, namely ethnographic participatory observations, including informal conversation as well as field notes, interviews, a focus group and a quantitative assessment of the physical environment. The observations formed the basis for the other methods, helping the researcher to get familiar with the setting. Data collection methods informed each other, allowing the validation of insights from different perspectives.

#### Ethnographic observations

To understand the daily life on the GCF and immerse in the setting, the first author, KR, lived at the GCF between June 2021 and August 2021, residing in a small house on the location. In total, 25 days of ethnographic participatory observations were undertaken by the first author. One of the team members, SS, completed an additional three days of observations to help discuss ideas and validate findings. In each of the three housing groups, three weeks were spent. During each week, two to three randomly chosen days were observed. Usually, observation periods lasted for 5 h, either during the morning (07:00–12:00), during the afternoon (12:00–17:00) or during the evening (17:00–22:00). In addition, one night shift (22:00–05:00) was observed. The goal was to get an overview of the life on the GCF. Observing actions and having informal conversations have been described as valuable tools to get insights into the habitual practice and can be more valuable than asking participants what they would have done in a certain situation (30). A few weeks prior to the start of the project, the first author was introduced via e-mail and posters hanging in each group. Before starting observations in a new group, the first author was personally introduced by the manager. The following observations usually started with a tour to get a sense of the daily life and the atmosphere (31). Afterwards, specific situations were chosen which seemed to be key moments during the day on the GCF. This could be mealtimes, indoor and outdoor activities or care- and other routines. Gradually, the first author became a part of the daily life at the GCF, working along the staff members. Informal conversations with residents, their visiting family members, staff members and volunteers were held in order to understand perspectives, opinions and lines of reasoning. During the observations, field notes were taken, helping to remember details observed during the day. Soon after, they were expanded into more elaborate notes. These included a physical description of where the observed situation took place, of the people participating, of the situation itself, including the role of each participant as well as conversations, and personal impressions about the atmosphere (32, 33). The field notes were regularly discussed within the team to determine potential follow-up moments to observe or questions to ask.

#### Interviews

As second part of the data collection, the first author held semi-structured interviews to get insights into the discourse at the GCF. In total, 24 interviews were held, with one interview including two participants. They were deliberately done after some weeks of observations and were informed by first insights gained there. They added more detailed opinions, reflections and background information than possible to gather during informal conversations during the ethnographic observations alone. From each of the three groups at the GCF, at least two residents, two family members and three staff members were interviewed. Additionally, other actors such as volunteers or activity coaches were included. Participants were purposefully sampled to reach maximum variation in demographic characteristics, relationship to the resident or functions. The first author invited them to participate after the first three weeks of participatory observations. After agreeing, a date for the interview was planned, where also the informed consent was signed. The baseline data of the participants is displayed in Table 1. Most of the interviews took place at the GCF, in various quiet locations chosen by the participant. Three interviews were held online. With residents in particular, the interviews were held in a relaxing atmosphere, for example while drinking a coffee in the private room. The interview guide for each participants group was developed after completing two weeks of ethnographic observations. First experiences and informal talks with the people met on location helped to identify relevant questions. The research team provided feedback for each interview guide. Questions were openly formulated and targeted, depending on the participant group, topics such as: “What do you like to do here during the day?”, “How would you describe your relationship with the residents here?” or “What is most important in the life of your relative?” Follow-up questions were asked to get a holistic and in-depth understanding of the participant's perspective. The interviewer stepped away from the interview guideline in case topics were identified which seemed especially important to the participant. The interviews lasted between 22 and 110 min and were audiotaped.

**Table 1:** Baseline characteristics of interview participants.

**Participant** **baseline**	***n =***	**Mean**	**SD**	**%**
**characteristics**				
Total	25			
Residents	6			
Age in years		86.17	2.91	
Women	5			83.3%
Family caregivers	7			
Age in years		61.57	9.96	
Women	5			71.4%
**Relationship with resident**				
*Child*	6			85.7%
*Spouse*	1			14.3%
Staff	12			
Age in years		50.33	12.43	
Women	10			83.3%
**Level of education**				
*Ongoing education*	1			8.3%
*Baccalaureate-educated registered nurse*	4			33.3%
*Vocationally-trained registered nurse*	3			25.0%
*Certified nurse assistant*	1			8.3%
*Nurse assistant/aide*	3			25.0%
Months employed at location		63.58	48.53	
Months working in function		89.58	121.26	
Years working in care		17.19	14.94	
Working hours per week		25.21	10.22	

#### Focus group

As third part of the data collection, a focus group was held with staff members in October 2021, after the ethnographic observation period. All staff members were invited by e-mail to join the focus group, which was planned for 2.5 h. The focus group was divided into three parts, starting with a short introduction. Thereafter, the staff members were invited to collect their favorite moments or activities during their work in a brainstorm session in smaller groups. After discussing results with the entire group, the staff members were again asked to come together in their groups. This time, they collected physical, social and organizational elements necessary to experience or do these moments. This was seen as a way to identify what is most important for employees on a GCF and the key components necessary for the functioning of this innovative care environment. A discussion leader, who steered the brainstorm and could ask further questions, led each group. The discussion leaders (*n* = 2) were members of the university, either directly involved in the present project (BdB) or involved in similar projects and carefully instructed. To capture the thoughts and ideas of the participants during the brainstorm sessions, the groups were provided with pens and large papers. During the focus group, the discussion leaders took notes about the conversations in the brainstorm sessions, which were converted into more extensive notes later. The notes that the staff members of each of the groups took during the session were photographed and digitalized by the first author afterwards. Additionally, the first author wrote a summary of the focus group, describing the key takeaways and the atmosphere.

#### Quantitative assessment tool

Lastly, the physical environment was assessed with the OAZIS-dementia tool, which was developed in 2015 for the Dutch long-term care setting (34). It consists of 72 items in the seven categories privacy and autonomy, sensory stimulation, view and nature, facilities, orientation and routing, domesticity, as well as safety. Items are scored on a 5-point Likert Scale from 1 (not at all) to 5 (completely). The tool was filled out by the first author (KR) at the end of the observation period in August 2021.

### Data analysis

The data sets of the ethnographic observations, interviews, the focus group and the quantitative assessment tool were analyzed in an iterative way. First, the ethnographic observations were analyzed by creating themes and coding (35). Insights gained there informed the analysis of the interviews and the focus group. The assessment tool was analyzed quantitatively. Iteratively, the findings from the different qualitative, as well as quantitative data sources were combined and discussed with the team. As relevant topics emerged in one data source, the other sources were searched to find insights on the same topic there. Like this, data sources informed each other, and linkages could be identified, as well as controversy (36). Each step of the data collection and analysis was noted down in a logbook, accessible for the entire team. This allowed to retrospectively follow the line of reasoning, ideas and discussion points (35).

#### Analysis of the qualitative data

In an iterative process, data analysis of the ethnographic field notes and interviews was performed in parallel with the data collection (32). For this, the observation notes were expanded into elaborate field notes and the interviews were transcribed verbatim by the first author. Family members and staff received a written summary of the interview for a member check (37, 38). Noting down first reflections, labels and connections in the data already collected helped the authors to focus on parts that seemed interesting and additionally, future data collection could be inspired with information from past observations and interviews (36).

After the data collection period ended, the data was formally analyzed with MaxQDA 2022. This included the observation field notes, interviews, as well as the information from the focus group. The analysis was guided by the conceptual framework developed by de Boer et al. (13). The framework describes the influence of the physical, social and organizational environment of a care organization on behavior and functioning of residents. In addition, inductive analyses were conducted, identifying any patterns or themes beyond the framework.

Data analysis followed the six-step model by Nowell et al. (35). The team members of the research team familiarized themselves with the data by repeatedly reading the different data sources. Afterwards, initial codes were generated using the observation data. Three team members (KR, BdB, SS) individually coded the same three randomly selected pages of the observation data and discussed their ideas afterwards to reach consensus. This process was repeated a second time and results were discussed with the entire team. Afterwards, KR coded ten randomly selected pages and discussed the results with the rest of the team. After agreeing on a suitable coding strategy, KR coded the remaining observation data, as well as the data from the focus group. The broad initial codes were based on the conceptual framework, and as the data analysis proceeded, more detailed codes were developed and sorted under each concept from the framework. This step had to be done repeatedly, as new, interesting codes emerged. Additionally, KR analyzed the interview transcripts by extracting the main messages in the form of quotes. They were systematically sorted by participant group and topic in a table. By directly comparing the quotes, an overall picture on each topic and participant group could be generated.

In the next step as described by Nowell et al. (35), the data was searched for patterns, linkages, but also controversy. From this step resulted a final phase of defining and naming codes and themes. At a certain point, no new information emerged from the data. Following, the themes were tested by returning to the raw data or by comparing codes and themes between the team members. Findings were summarized, followed by a thorough discussion of the data among the entire team to determine whether the interpretation seemed complete and credible. The last step as reported by Nowell (35), producing the report, was done throughout the entire data collection and analysis period and included descriptions of the context, and the reasoning for theoretical, methodological or analytical choices.

#### Analysis of the quantitative data

In total, 340 points can be reached on the OAZIS-dementia tool. The 72 items are distributed over seven categories and scored on a 5-point Likert scale (34). For each category, the points reached were summed up and an average value was calculated by dividing them by the total possible amount of points. Subsequently, a final average score was calculated in the same manner (14).

### Ethics and consent

All legal representatives of residents, as well as staff members received information about the study and a consent form for participation via e-mail and post. Legal representatives provided informed consent for themselves, as well as the resident. During the observations, the first author paid close attention to signs of discomfort of residents. For example, the staff member involved in the care situation asked the resident beforehand whether the first author is allowed to join. In case the resident expressed any signs of distress during the care situation, the first author withdrew her attendance. The interviews with residents were only held after getting assent from the participant (39). Beforehand, a staff member asked them whether they would like to have a conversation with the first author, who will be asking them some questions. Only when agreeing, the first author approached the resident. All data was anonymized. The GCF was asked whether its name should be publicly disclosed in this study. The study was approved by the ethical committee METC Z (No. METCZ20210097).

## Results

The analyses revealed a conscious harmonization of the physical, social and organizational environment at the GCF. With 314 of 340 total points, the physical environment of the GCF scored high on the OAZIS-dementia tool. This indicates a suitable environment for people living with dementia. The observations confirmed that the architectural design of the physical environment with its indoor and outdoor spaces opened up possibilities for residents to move freely and be active. At the same time, the organizational environment was explicitly designed in a way supporting and stimulating its use with suitable organizational processes. This in turn opened up possibilities within the social environment, fostering for example social encounters. This well-balanced interrelation of the three environments seemed to benefit not only residents, but also their family members or other visitors, as well as staff members and the management.

From the analysis of the qualitative data, four themes resulted which were identified as crucial during the daily life at the GCF. These were stimulating the senses, engaging in purposeful activities, sharing responsibilities and creating a community in a new home. They serve as examples illuminating the interrelatedness of the physical, social and organizational environment.

### Stimulating the senses

As part of the vision of the GCF, a strong focus was put on a stimulation of the senses and activity. Realizing this, the managers designed the physical environment in a way that activated staff and residents in a natural way. Mostly built on ground level and covered by lightly painted wood, the buildings of the GCF naturally blended into the gardens and animal meadows surrounding them. The resident rooms of each group were located apart from the common house, separated by a small garden. In the garden, a mix of trees, bushes and different colorful flowers grew, attracting butterflies and bees. This also provided residents with more advanced dementia with visual and audible stimulation, as described in the following observation note made in one of the groups:

After the coffee, the staff member Jan picks me up from sitting at the table with the residents to quickly ask me about my first impressions. As we walk through the garden of the group, he tells me that he really likes that the residents have to walk through it to get to the common house as it gives people stimuli. He tells me about a resident who doesn't talk much, but on the way through the garden, she stops here and there and shows him a flower, or a bug, or something else catching her eye. (Fieldnote 10)

Several other architectural design choices encouraged daily activity and sensory stimulation. A daily ritual on the GCF was bringing away the garbage and the leftovers from the kitchen. Each evening after dinner, staff members collected a number of residents to participate in this household task. The containers for mixed garbage, plastic and glass were deliberately placed apart from the groups, each at a different end of the location. The leftovers from the kitchen were brought to the pigs, again located a few meters apart from the groups. The resulting evening walks not only allowed residents to contribute something useful to the community, they also resulted in daily exercise. The following observation note provides an example on how the design of the outside environment has the potential to turn a household activity into an extensive walk with a number of different experiences on the way:

After collecting five residents, we start our walk to the pigs to bring them some leftovers from the food and the potato skins which resident Eline produced today. On our way back, we take a little extra round and turn into a path between two meadows. We come by the horses, who are standing at the fence. Resident Maria starts telling me that she also rode horses when she was younger, and we look at the small ponies eating grass. One of the large horses smells our hands curiously. We continue our walk through the two meadows until we reach the path under the trees. Here, we pass the “singing hut”, a wooden hut where one can sit down and turn on some music, while enjoying the view on the horses. Maria climbs up the few steps and looks inside, then comes down on the ramp on the other side, waving at us. Next, we come by the lake where the playground for children is. We make jokes how another resident, Jacob, can jump on the trampoline if he wants, and Lydia makes music on the outdoor music instrument with her walking stick. After some minutes, we walk back through the gate towards the common house of our group. (Fieldnote 187)

This example shows how the physical environment has the potential to alter the social environment substantially, when designed consciously. In this case, the physical environment of the GCF provides the opportunity to turn a household task, like bringing away the leftovers from the kitchen, into an interesting and fun group activity, which naturally incorporates exercise. Walking to the pigs and back to their houses, residents had diverse experiences during which all senses were stimulated. Furthermore, residents were encouraged to talk about their past when seeing the horses. The following quote illustrates how placing several locations, necessary for the daily life, far apart from each other, was a conscious choice made by the managers upon building the nursing home:

“So one of the things we also took into account in the construction here is that, well, you have to build and furnish in such a way that it is logical that you go outside. You have to go outside here whether it is storming or raining or very hot, so in that sense we strongly believe that change in the care really starts with a different way of building. And not only that you indeed have facilities and have a garden and butterflies outside, but also that you use them as an employee. And we even think that you have to further enforce that because we say bring away garbage, that's over there, they have to bring something to the animals that's over there, or they have to pick up something in the country house which forces the employees to do that too. And now it's no longer a discussion here, everybody goes outside and likes to go outside (…)” (P11, translated from Dutch)

This quote from the managers highlights the role of the physical and the organizational environment in stimulating to go outside. According to the managers, the architectural design of the outside environment can provide opportunities for activity. At the same time, it has to be designed in a way that “forces” staff to also do so.

While the design of the physical environment opened up possibilities for stimulation and activity, it also provided the opportunity to withdraw to places with less sensory stimulation. Living in a large group sometimes seemed to be challenging for some residents. The common houses were split up into a large kitchen and two living rooms. Together with the resident houses, as well as the outside environment, residents had several spaces where they could spend their time. This also provided residents the opportunity to withdraw from the group when they wished to be alone, or to be together in smaller or larger groups. During an evening observation, the first author was sitting outside on the terrace with residents and staff members. As the large group seemed to put pressure on one of the residents, a staff member took a small walk with her to a Hollywood swing a few meters apart to help her calm down:

In the circle of residents and staff members, I sit next to Elizabeth. She seems stressed – she changes her focus very quickly, looks at different people, in between, she closes her eyes as if she wanted a break. She turns to me and says, “this is really bad”. I quickly understand that she doesn't like to be with that many people. Staff member Anna, sitting in the circle with us, also notices that she is stressed and says: “There are too many people, right? This stresses you out” and Elizabeth nods, closing her eyes. Anna gets up and takes her arm, and together, they go for a walk. I see them sitting down on a Hollywood swing, and Anna calmly talks to Elizabeth, pointing at something she sees. After a while, they come back, and Anna accompanies Elizabeth to the inside of the common house. She seems calmer now and smiles at us when they walk past us. (Fieldnote 72)

This example not only illuminates the importance of the design of the physical environment in providing possibilities to retract. It also illustrates the critical role of staff in identifying, and resolving moments of uneasiness among residents. In this case, the staff member felt that a resident was uncomfortable, although the resident herself could not clearly state what her feelings were. Supported by the other staff members keeping an eye on the remaining group outside, she could go for a walk to calm down the resident. Being able to leave the group to help one resident relax calls for a strong feeling of collaboration among staff. At the GCF, a strong organizational culture persisted, where tasks were often shared among staff and where the well-being of residents was considered more important than potential tasks to be completed.

Concluding, the physical environment of the GCF opened up possibilities for as well sensory stimulation and activity, as the possibility to detach from too much sensory stimulation. The organizational environment played a crucial role in designing the physical environment upon building the nursing home, as well as identifying resident's needs and guiding behavior. Only in combination, the physical and the organizational environment can exercise its potential and create beneficial effects in the social environment, for residents, as well as staff members.

### Engaging in purposeful activities

At the GCF were countless possibilities to engage in activities. Outside, residents could for example feed the animals or care for the garden. Inside, residents could help in the household with folding laundry, chopping vegetables for dinner or setting the tables. A common feature of these activities was that they benefitted the group or the nursing home as a whole. Other than merely taking a walk, residents could take a walk to feed the animals, which added a purpose to the activity and benefitted the community.

On the one hand, the physical environment was designed in a way that offered the possibility to engage in nature-based, or other purposeful activities, as for example household chores. Each group had for example own chickens right next to the common house who had to be fed daily. Often, this was done by residents, who were not only active physically, but also had a daily goal. It seemed as if many of them enjoyed being useful for the group, contributing something and not only receiving care, but also caring for something themselves. In addition, the common houses were designed to promote a home-like feeling and stimulate the participation in household chores. Each group had an own kitchen with a large table where staff members planned and cooked each meal themselves. This gave residents the possibility to be involved in choosing and preparing the food. At the same time, the smell of freshly cooked meals activates the senses and makes a place feel like home, as one family member noted:

“I think they first have to build the nursing homes differently, (…) often the kitchen is central and the food is brought there. Here they cook themselves so then you have that home-like feeling again. When you come in here you immediately smell the food, so yes that is just the hominess” (F5, translated from Dutch)

This quote by a family member highlights the positive effects of cooking within the resident groups, as the smell of a freshly cooked meal contributes to a home-like feeling. At the same time, cooking within the group offers residents the possibility to participate in the activities in the kitchen and hence to be active and contribute something to the community.

The observations highlighted the important role of staff when involving residents in activities around the household. At the GCF, a strong feeling of living here together and sharing a household persisted among staff and residents. By regularly spending time within the groups, the managers explicitly encouraged staff members to think of every task to be completed as an activity for residents. Staff members seemed to have internalized this vision, exemplified in the following observation:

After I finish my coffee, I walk inside to the kitchen to put my cup in the dishwasher. Staff member Hanna sees me and tells me that I can just leave the cup on top of the counter, because residents often help cleaning the kitchen and they will later put the cup in the dishwasher. (Fieldnote 24)

In this example, the staff member purposefully reserved work for residents by hindering the first author to put her own cup in the dishwasher. During the observations, the first author also often noticed how staff members had a special way of motivating residents. For example, instead of asking residents whether they could fold the laundry, they asked whether they would be so kind to help them with folding the laundry. It seemed like residents were usually keen and happy to help the one asking and immediately joined the task. Moreover, staff members often created a fun and inviting atmosphere during these activities, illustrated by the following observation note:

After cleaning the dishes, the hostess asks three residents sitting at the kitchen table to help her dry. She hands Anna, Eline and Gerda a towel and they start drying the cups. Eline seems to like helping with household tasks; I saw her peeling potatoes a lot, drying dishes or folding clean cloths and towels. Another resident, Jacob, comes to the table and the hostess asks him whether he would like to help, too. He agrees and also receives a towel and joins the ladies. I sit on the terrace with some other residents and hear the people in the kitchen sing some old songs together. Jacobs loud, deep voice and the hostesses higher voice reach us at the terrace. (Fieldnote 236)

The participation of residents in a common household task has the potential to become a social activity where everyone involved benefits. Not only the residents, who contribute something and are active cognitively and physically, while enjoying to sing, also the staff member who can share the task benefits.

In conclusion, the GCF with its indoor and outdoor environment provided the residents with a variety of possibilities to be active in a purposeful way. At the same time, staff members played a crucial role in motivating residents in the right way, addressing their wish to help. Involving residents in activities evolving around the household, the animals or the gardens created a community feeling, as residents contributed something to their group or the nursing home as a whole. Often, these activities became a social event, with staff and residents benefitting similarly.

### Sharing responsibilities

According to the managers, life at the GCF should be as normal as possible for residents. They were encouraged to take own decisions, do what they liked and move freely on the location. One important element for realizing this were open doors. Residents could move independently between the common house and their rooms, located in small houses separated from the common house by a garden. Being outside every day, residents experienced the seasons, different weather, and had a feeling of “going somewhere” and “coming back home”. Animal meadows surrounded the houses of each group and served as a natural barrier to the rest of the location and the village. However, the gates to the location were always open, allowing residents to not only take a walk in the garden of their group, but also freely access the three-acre location with its animals and gardens. Valuing the dignity and independence of residents, there was no explicit emphasis on constantly keeping an eye on them. Still, several elements within the physical and the organizational environment supported residents' freedom, and, at the same time, residents' security. Within the physical environment, this were architectural and technological measures, within the organizational and social environment, sharing responsibilities played a key role.

An architectural measure was the built-design of the common houses, with their bottom deep windows, which could be opened as doors. Being built on the ground floor and having glass doors on all sides of the house had several advantages. First, the windows provided natural light for the indoor environment. Second, residents spending time in the kitchen or living rooms could watch the outdoors with its nature, animals or people coming by. Third, residents could easily access the outdoor environment from several sides of the house. Lastly, staff members could easily oversee events taking place both inside and outside.

Furthermore, several technological measures, such as sensors, supported the security of residents. Specifically relevant during the day were the sensors applied to the gate, separating a group from the rest of the location. According to the managers, one to two residents per group had a sensor applied to their clothes. Whenever a resident with such a sensor walked through the gate, the telephones of the staff members rang. This allowed them to follow their tasks without having to constantly watch the gates. As the telephone rang, they quickly checked who walked in- or outside and could decide whether this person needed assistance.

Despite these architectural and technological measures enabling residents to freely move on the location, the sharing of responsibilities between the management, staff members and the family of each resident was a crucial factor enabling residents' freedom. Before moving into the GCF, the managers informed the family members of a potential new resident about the open door policy. Consequently, only residents moved into the facility, whose family took the informed decision in favor of open doors. According to the managers, the families valued the positive effects resulting from the freedom higher than the potential risks. Knowing that families were in favor of open doors and aware of the risks coming with it, staff felt more secure to allow residents to take a walk and be active on their own. This substantially increased the time residents spent outside. Nevertheless, the risk of residents getting lost is an undeniable factor in nursing homes for people with dementia. The management of the FCG indicated that residents walking beyond the perimeter of the locations only occurred a few times in the last years. In line with the wishes of staff and families, the managers strongly contradicted closing the doors of the GCF because of single cases, which would result in negative consequences for all residents. Instead, in the few cases where residents tended to walk beyond the perimeters of the location, they brought together the family and staff members to jointly decide how to prevent such incidents in the future. The following quote from the managers shows how a family assessed the situation in a case where a resident liked to take walks outside the location and might get lost:

“Well, that is quite exciting, also for us - we have very well discussed with the family, how do we deal with it? And the family is really agreeing. The family also wants someone to have the freedom to walk, and takes the risk; well that could also go wrong in a very bad case, right? (…) That requires talking to family and also in the team: how do you deal with that? Because it is a kind of balancing act, isn't it? Because it is not like let them go and you do not have to watch them, you have to watch them!” (P11, translated from Dutch)

This quote exemplifies the close collaboration between the management and the families. The fact that family members were aware of the risks and could bring in their own wishes concerning the measures taken relieved staff of responsibility. During the observations, also staff widely seemed to value the freedom, which residents had, and accepted the risks coming with an open-door policy. The following quote from a staff member represents the common belief on the GCF that the freedom outbalances the risks of getting lost:

“Well, whether you work in a nursing home or on the care farm, risks are everywhere. And the risk of someone leaving [the location] is there! And it's fine that it's there! Because in order to make this possible, you have to have some kind of acceptance that it can happen, and I wouldn't want to change that. I would find it terrible if the doors would close (…) because I think there are bigger risks than when they are open. The moment someone can no longer get out, someone will think of how he or she can get out. And then they go under or climb over the fence and that brings more risks with it, than that someone can walk out of the fence and I get a ring and see hey, someone walks out of the gate.” (P9, translated from Dutch)

The interviewed staff member seems highly positive about the open door policy and even considers the risks of closed doors as more severe than the risk of open doors. The fact that the staff member does not share concerns regarding the responsibility of a lost resident indicates a strong organizational support and cohesion of involved parties.

In conclusion, the example of open doors illustrates how a close collaboration between the social environment, i.e. the families, and the organizational environment, i.e. staff and management, can have positive effects for residents. Together with architectural and technological measures taken to increase oversight of the location, the freedom of residents can be fostered, who might otherwise be restricted due to security reasons. This interrelatedness of the three environments opened up possibilities for residents to engage in activities within their group, or even on the entire location.

### Creating a community in a new home

The observations revealed a home-like atmosphere at the GCF. Creating a sense of home and having as much of a normal life as possible was one of the most important goals of the managers. They lived next door and were often present on the premises. In the first years after opening the location, both worked in the groups themselves, which facilitated the transportation of their vision by being a role model. Until today, they regularly spent time in each group to collaboratively create a community, support the staff members in their daily work, and to be able to correct habits not in line with their vision. The following situation illustrates how the managers actively corrected habits in order to create a more home-like atmosphere: One day, after starting the observation period in a new group, the first author realized that this group used plastic cups during lunch, instead of glasses like the group before. A few weeks later, during the interview with the managers, they stated the following:

P11: “Then I see for example at a group suddenly that they drink with colored cups, like plastic colored cups. We don't do that at home either, we don't drink from a plastic cup! (…) that is an example of how it is probably more practical or handy and you can stack it (…).” (P11, translated from Dutch)

P12: “This is often the case in health care; we don't want the convenience of the organization to be the guiding principle, the guiding principle is that you just live your life the way you do. And if you drink out of a glass, you drink out of a glass, that's what you did at home, then here too. (…) And the care sector is very often used to working very much from an organizational perspective or from an efficiency perspective and that is not the same as creating the best atmosphere.” (P12, translated from Dutch)

This quote illustrates how both managers preferred atmosphere to efficiency. This included details like the use of glasses instead of plastic cups, but also that residents used the same dishes as staff members. This, according to them, supported a home-like, community feeling and showed respect for the residents and their way of living.

At the same time, a cozy atmosphere was also created by the design of private and communal areas. Aimed at seeming more like a vacation park than a nursing home, the buildings of the GCF were mostly built on ground level and covered by wood. The furnishing of the indoor environment further supported a home-like atmosphere. Residents were not only invited to furnish their own room individually, they could also bring for example art and furniture for the common areas. Possibly attributed to the fact that residents contributed to the decoration of the common house and helped with the household, a strong sense of being part of the community became apparent. Often, residents intrinsically picked up a pillow lying on the floor, swept the terrace, cleaned up leaves from flowers, or had a precise idea of how the lace should be folded. This is illustrated by the following observation made after lunch:

After we cleaned up the table – again, all residents helped – we put the flowers back on the table. Some old leaves fall down on the floor and resident Margot directly reaches down to pick them up. She sees some more of another bouquet and walks over to also pick those up. “Very nice, thank you” I say and she looks at me, smiling and saying that she likes it clean. Resident Willeke joins our conversation and says that she also hates it when the white lace is thrown in some corner while the table cloth is on the table during eating times. “Yes, Willeke really doesn't like that!” Margot laughs. “We always fold it nicely and put it over the sofa.” (Fieldnote 272)

Additionally, a friendly and inviting culture persisted at the farm, described by both family members and staff during the interviews. A family member for example stated the following after being asked how the relation with the staff is:

“Yes also like that, just loving, warm, yes, understanding. Also know who you are. Know that you have been on vacation when you come back. You actually- when I come here it is like coming home again. Really coming home. It is a kind of second home.” (F2, translated from Dutch)

The observations showed that, indeed, many family members came to visit. Staff members always made sure that they felt welcomed and comfortable by offering them a coffee and a seat, and asking how they are. Family members visiting during mealtimes were invited to join the meal along with residents and staff members. Enjoying the welcoming atmosphere, many family members spent the time with their loved one not in the private room but within the group, having conversations with the other residents as well. Indirectly, this relieved staff members from a part of their supervising tasks and added to the social interactions of residents. Knowing that a family member was keeping an eye on the group sitting on the terrace or in the living room, staff members could focus on residents in other rooms or spend more time with those needing individual attention.

In conclusion, the physical environment, as well as an organizational vision exercised by management and staff created a home-like atmosphere at the GCF. This resulted in residents feeling a sense of ownership, intrinsically keeping their common house clean. Furthermore, family members felt welcomed and by staying within the group, indirectly relived staff members by watching out for residents.

## Discussion

This study explored the care environment of GCFs for people with dementia. Four central themes could be identified: stimulating the senses, engaging in purposeful activities, sharing responsibilities and creating a community in a new home. In comparison with traditional care, GCFs are radically different in the physical, social and organizational environment. The findings accentuated the necessary high degree of interrelatedness of the three environments, each one supporting the others. Designed in line with the organizational vision, the physical environment provided opportunities to stimulate the senses, activity and social encounters. The organizational environment played a key role in activating residents and hence optimally using the physical environment. By sharing the responsibilities and creating an inviting atmosphere, the social network of residents was included into decisions and in the daily life on the GCF. Consequently, residents, their families, staff members and the management benefitted from social interaction, activity and collaboration.

### The crucial role of the management

The findings of this study highlight the crucial role of the managers of the GCF in paving the way in the physical, social and organizational environment. Based on their vision, they designed the three environments in a way that each one increased possibilities within the others. As the GCF was newly built, the physical environment was planned by the managers of the GCF. Hence, its design, including the buildings, indoor decorations and outdoor facilities was a conscious organizational choice, intended at creating possibilities for stimulation, activity, social interaction and a home-like atmosphere. Consequently, the physical environment is, to a certain degree, dependent on organizational choices.

As this study showed, the design of the physical environment substantially shapes the realization of organizational goals and visions. This is in line with previous research, indicating that the design of buildings is correlated with a higher quality of life of residents (18). Furthermore, research has shown that residents' social life and engagement in activities depend on a dementia-sensitive environment (40, 41), and that the physical environment forms the basis for what residents perceive as home-like (42). Additionally, GCFs actively use nature to provide naturally-emerging, purposeful activities. Gardens are suggested to reduce agitation in people with dementia (43) and may have positive effects on psychological well-being and loneliness (44, 45). Furthermore, evidence is accumulating that residents' interaction with animals, e.g., animal-assisted activities or animal-assisted interventions, could have positive effects. For example, positive emotions and social interactions were registered more frequently and longer (46). Additionally, a systematic review showed that social functioning was improved across all severity levels of dementia (47) and other findings suggest that the progression of agitation or depression could be slowed down (48).

At the same time, this study shows that the day-to-day organizational processes the social context are equally important as a suitable physical environment. Staff members using the physical environment in the right way and including residents and family into day-to-day activities, are essential (43, 49). This indicates a need of nursing staff to adapt their way of working to encourage residents to participate in daily activities (50). Here, too, the management of an organization plays a crucial role as they can actively support staff members in executing the vision by creating a suitable organizational environment. Previous studies have found for example that shared values and supportive leadership for staff help in setting priorities and improve the delivery of person-centered care (25, 51, 52). This study builds on these results and shows that the underlying organizational processes, including for example the leadership style, rules and routines within an organization substantially shape the way, in which daily life is organized and ultimately how care is delivered. An example is to leave staff members flexibility in deciding the daily time schedules. Sometimes, it takes longer to include residents into tasks and a culture following strict routines might hinder the daily engagement of residents. Furthermore, the management can pave the way in the social environment by creating a positive atmosphere. Establishing a social environment that also builds and fosters activity, collaboration and a positive atmosphere ultimately benefits all groups involved (53, 54). This study showed how this could also feed back into the organizational environment by relieving staff members of supervisory tasks. Rethinking dementia care by radically altering the physical, social and organizational environment to better meet the needs of residents indicates a rebellion-like mindset of the founders (27, 55). This includes creating an environment, which is focused on seeing the person beyond the disability, instead of the convenience of the organization.

### Collaboration between management, staff, residents and families

Building on the described preconditions in the physical, social and organizational environment, the atmosphere on the GCF was characterized by a sense of “doing everything together”. On the one hand, this was attributed to the active collaboration among staff, management and families in decisions concerning residents. Because families were aware of potential risks and took the informed decision of accepting these, staff felt more secure to allow residents to use the outside environment on their own. Furthermore, the open and inviting atmosphere at the GCF encouraged family members to spend time within the group. Research has shown the importance for residents to preserve their former social network and that their family or friends feel welcome in the nursing home, for example through nurses greeting them and offering them a seat and a coffee (56). Forming a community of residents, staff, families and the management builds on the principle of relationship-centered care (57, 58). Relationship centered care stems from a more inclusive approach to dementia care, recognizing also on the social network of the person with dementia. The initial focus on couples has gradually expanded to the wider family and beyond; consequently, the focus of care provision is not only on the person with dementia. Instead, it includes the well-being of family and the reciprocal ways in which people with dementia also can give back (59).

Valuing the ways in which residents can also give back indicates the second reason for a feeling of “doing everything together”, which is the active encouragement of residents to contribute to the community with their individual skills. One of the key goals of the GCF was the inclusion of residents into purposeful activities, such as household chores. Residents were consulted for the selection of meals and the preparation of such, as well as involved in bringing away the trash at the end of the day or feeding the animals. This contradicts a more traditional view where the staff member takes over as many tasks as possible for the resident (60). Previous research has found that a key determinant of the quality of life of people with moderate to advanced dementia was contributing to the household (61) and generally giving a meaning to life (62). Explicitly taking a resident perspective and designing a nursing home supporting their needs and wishes indicates a culture change within nursing home care (63, 64). This includes the creation of environments that “allow the person with dementia to be an active participant in everyday life rather than a passive recipient of care” [(64) p. 186–7] and is in line with Kitwoods theory of person-centered care (65). The basis is a positive attitude toward the person with dementia, his or her unique personality and maintaining and strengthening of the personhood. Kitwood (66) emphasizes the necessity to satisfy the psychological needs of people with dementia, as this is the prerequisite to function as a person. This can be translated into practice by not looking at what people with dementia cannot do anymore, but instead embracing their interests, their pleasures and the use of remaining capacities (67). On the GCF, staff actively used and fostered the abilities that residents still had and often motivated residents to use their skills to contribute to the community in a meaningful way. Interestingly, this seemed to result in a feeling of a shared household, as there were also moments where residents intrinsically for instance arranged the flowers or put the tablecloth on the table. Taking own initiative and contributing to the household indicates that residents, too, felt that they were “doing everything together” and potentially contributed to their sense of being at home in the nursing home.

The fact that residents, their families and staff members equally seemed to benefit from collaboratively doing life, seemed to preserve the initial vision the managers implemented. In the seven years since the foundation of the GCF, the vision seemed to be transported between generations of residents, families and staff members. Only with minor corrections, the managers succeeded to continue delivering the care they defined upon founding the nursing home. This shows how radically rethinking dementia care requires passionate leaders, transporting their vision and paving the way in the physical, social and organizational environment to initiate change. When implementing new ways of working, they ultimately might prove to benefit all stakeholders involved. This can create a valuable partnership, where staff members enjoy their work, families feel appreciated and residents with dementia can be valuable contributors to the community.

### Methodological discussion

The present study provides an in-depth exploration of the care environment of an innovative care concept. A care organization consists of infinite preconditions, processes and uncertainties, which makes a complete assessment impossible. In this context, the combination of diverse methods can be considered as a strength, because it enabled a detailed exploration of the complex environment, including the perspectives of involved stakeholders. Corresponding to a constructivist approach to data collection and analysis, the researchers inherently are subjective (68) and previous experiences might influence data collection and analysis. This requires reflexivity from the researchers. Within the research team, the experiences made, the data collected and the analyses were regularly discussed to include other perspectives. Furthermore, involving another team member into data collection validated insights. A common problem within qualitative research is the Hawthorne effect, which describes the phenomenon of participants behaving differently because they are studied (69). The long time frame of several months was chosen to mitigate this effect, as staff, residents and families became used to the presence of the researcher.

## Conclusion

In conclusion, the way in which a care organization is designed significantly impacts residents' daily life and their mental, physical and social functioning. To better meet their individual needs, GCFs have radically altered the physical, social and organizational environment. By aligning the three environments, and using each one to support the others, the GCF created four powerful topics, defining daily life. These were stimulating the senses, engaging in purposeful activities, sharing responsibilities and creating a community in a new home. This study showed that in order to successfully innovate long-term care, leaders are needed who rethink existing ways of care delivery. This begins with sensing opportunities and transforming the physical, social and organizational environment to support their staff seizing these opportunities. The physical environment needs to be designed in an encouraging way, stimulating activities. A social sphere has to be created where everyone is welcomed openly and where the entire network of the organization thrives through collaboration. Finally, to successfully lead change, organizational processes have to fit the vision, and support residents, staff, families and management equally in executing the vision. Creating an environment where all stakeholders of a care organization benefit leads to a collaborative, productive way of delivering care to those in need.

This study contributes to the research field by providing an example on how joint alterations in the physical, social and organizational environment of a care organization can lead to sustainable changes, benefitting all stakeholders. Learnings from GCFs are possibly transferable to other care settings, facing difficulties in bringing about change. With further research, the role of the organizational environment could be explored in more detail, identifying strategies actively supporting a culture change within long-term care organizations. Furthermore, insights into barriers and facilitators in doing so might help nursing homes to adapt to new ways of delivering long-term care.

## Data availability statement

The original contributions presented in the study are included in the article/supplementary materials, further inquiries can be directed to the corresponding author.

## Ethics statement

The studies involving human participants were reviewed and approved by Medisch Ethische Toetsingscommissie Zuyderland en Zuyd Hogeschool (METCZ20210097). The patients/participants provided their written informed consent to participate in this study.

## Author contributions

This project was designed by KR, BB, and HV. KR collected the data with help of SS. KR conducted the main analyses and wrote the manuscript, both with assistance of BB, HV, SS, and JS, who provided feedback and guidance. All authors contributed to the article and approved the submitted version.

## Funding

This study was funded by Meandergroep Zuid Limburg, Maastricht University and the Novartis University of Basel Excellence Scholarships for Life Sciences.

## Conflict of interest

The authors declare that the research was conducted in the absence of any commercial or financial relationships that could be construed as a potential conflict of interest.

## Publisher's note

All claims expressed in this article are solely those of the authors and do not necessarily represent those of their affiliated organizations, or those of the publisher, the editors and the reviewers. Any product that may be evaluated in this article, or claim that may be made by its manufacturer, is not guaranteed or endorsed by the publisher.
